# Homotypic Cell Membrane-Cloaked Biomimetic Nanocarrier for the Targeted Chemotherapy of Hepatocellular Carcinoma

**DOI:** 10.7150/thno.34837

**Published:** 2019-08-12

**Authors:** Xiaojun Liu, Yingxue Sun, Shushen Xu, Xiaonan Gao, Fanpeng Kong, Kehua Xu, Bo Tang

**Affiliations:** College of Chemistry, Chemical Engineering and Materials Science, Key Laboratory of Molecular and Nano Probes, Ministry of Education, Collaborative Innovation Center of Functionalized Probes for Chemical Imaging in Universities of Shandong, Institute of Molecular and Nano Science, Shandong Normal University, Jinan 250014, P. R. China.

**Keywords:** homotypic, biomimetic, nanocarrier, cancer cell membrane, hepatocellular carcinoma

## Abstract

**Goals:** Hepatocellular carcinoma (HCC) has been reported to be the third most common malignant tumor and has the highest rate of mortality. To increase the chemotherapy efficacy of HCC, a drug delivery system featured with desirable active targeting ability, delivery efficiency and immune evasion is in high demand.

**Methods:** We have developed a drug nanocarrier by utilizing a homotypic cancer cell membrane for targeted chemotherapy of HCC. Structurally, the homotypic HepG2 cell membrane was used as the cloak, and a poly (lactic-*co*-glycolic acid) (PLGA) nanoparticle as the core, resulting in the nanocarrier **HepM-PLGA**.

**Results:** The **HepM-PLGA** nanoparticles exhibit excellent targeting ability toward HepG2 cells. Doxorubicin (Dox) carried by **HepM-PLGA** possesses high delivery efficiency and a remarkable *in vitro* therapeutic effect. In *in vivo* experiments, **HepM-PLGA** delivers Dox directly to the tumor lesion of nude mice, and tumor volume decreases by approximately 90% after treatment.

**Conclusion:** We have developed a drug nanocarrier by utilizing a homotypic cancer cell membrane for targeted chemotherapy of HCC with excellent active targeting ability. This biomimetic platform not only effectively treats HCC but also provides a sound strategy for the treatment of other cancers *via* changes in the corresponding homotypic cancer cell membrane.

## Introduction

Hepatocellular carcinoma (HCC), as common malignant tumor, has high rate of mortality 1-4. Chemotherapy using small molecule drugs carried by various types of drug delivery systems serves as one of the therapeutic modalities for HCC 5. Recently, literatures extensively explored the delivery system with focus on nanomaterials, including Au nanoparticles and polymer nanoparticles such as poly (lactic-*co*- glycolic acid) (PLGA) with functionalization *via* various targeting ligands like antibodies, nucleic acids and peptides 6-13. However, the clinical results of drug-carrying nanomaterials were still significantly restricted, since the poorly limited passive targeting ability through enhanced permeability and retention (EPR) effect 14-16, and relatively short circulation time as well as premature drug leakage *in vivo* resulted by the intrinsic large specific surface area of the nanomaterials 17,18. Moreover, nanomaterials are prone to be engulfed by macrophages, thereby triggering weak immunocompatibility 19. Hence, it is imperative to develop drug nanocarriers with excellent targeting and immune escape capabilities through suitable approaches to enhance the cancer chemotherapy.

In this regard, the biomimetic strategy with utilization of natural cellular membranes for nanoparticle surface functionalization was taken into consideration 20. To date, natural cellular membranes, including red blood cell 21-23, platelet 24, stem cell 25 membranes and cancer cell membrane 26,27 have been used to establish functionalized nanoparticles. Among these cell membranes, cancer cell membrane attracts our attention due to their unique homotypic aggregation and immune escape abilities 28-30. Both the formation of the primary tumor mass and the tumor metastasis relies on the homotypic aggregation behavior, since the surface membrane proteins of the cancer cells account for the homotypy 31,32. To this point, we hypothesize applying homotypic cancer cell membrane as cloak for nanomaterials to generate desirable drug nanocarriers by suitable strategies, as the surface protein interactions of the homotypic cancer cell membrane endow the nanocarrier with excellent homotypic targeting ability and remarkable immunocompatibility 33-35. The designed nanocarrier will be able to prolong the circulation time and improve the *in vivo* drug delivery efficiency 36-40. So far, it has been rarely reported to improve the chemotherapy efficacy of HCC by making use of the homogenous aggregation ability of HCC cell membranes.

**Scheme 1:**
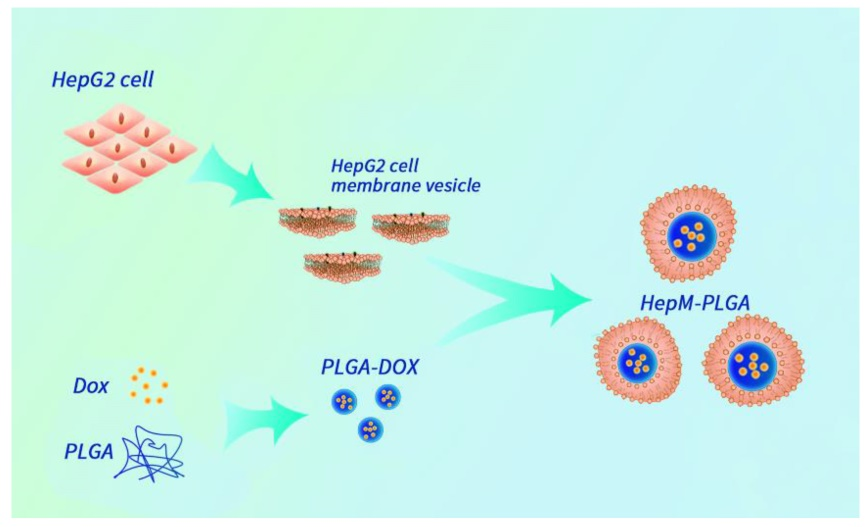
The design strategy of the cancer cell membrane-biomimetic drug nanocarrier **HepM-PLGA**.

Herein, we designed an HCC cell membrane- biomimetic drug nanocarrier with the aid of the homotypic aggregation of cancer cells for treatment of HCC. The nanocarrier platform **HepM-PLGA** was constructed by HCC cell membrane coated PLGA nanoparticles, and doxorubicin (Dox) was chosen as the model anticancer drug to be effectively encapsulated into the **HepM-PLGA** nanoparticles with high drug loading efficiency (Scheme 1). The **HepM-PLGA** targets HepG2 cells well, and simultaneously Dox was carried by **HepM-PLGA** directly to the tumor region to dramatically reduce the tumor volume. Hence, the biomimetic **HepM-PLGA** platform offers new perspective as powerful drug delivery system for cancer chemotherapy in the future.

## Results and Discussion

### Preparation and characterization of HepM-PLGA

**Figure 1:**
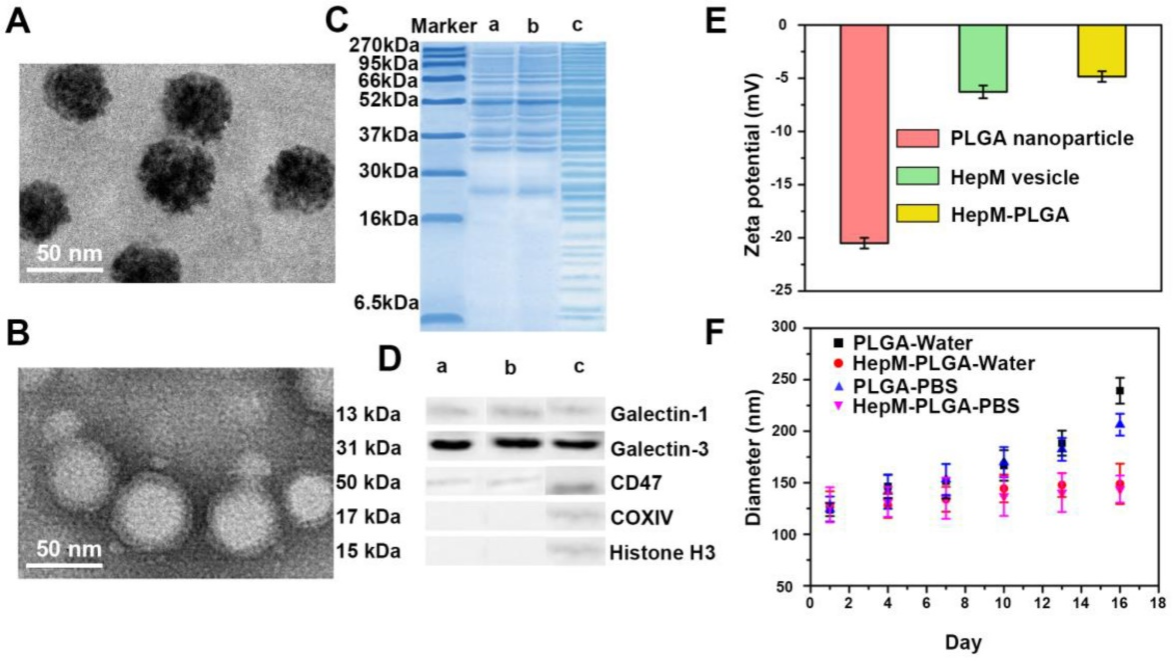
Preparation and characterization of **HepM-PLGA**. TEM image of (A) bare PLGA nanoparticles and (B) **HepM-PLGA** nanoparticles. (C) Gel electrophoresis analysis of (a) **HepM-PLGA** nanoparticles, (b) HepG2 cell membrane vesicles and (c) HepG2 cell lysis solutions. (D) Western blot analysis of (a) **HepM-PLGA** nanoparticles, (b) HepG2 cell membrane vesicles and (c) HepG2 cell lysis solutions. (E) Zeta potentials of bare PLGA nanoparticles, **HepM-PLGA** nanoparticles and HepG2 cell membrane vesicles. (F) Stability of PLGA nanoparticles and **HepM-PLGA** nanoparticles in water and PBS.

PLGA nanoparticles were first synthesized and the **HepM-PLGA** was prepared with the top-down method. HepG2 cell membranes obtained from HepG2 cells were used as the cloak to functionalize the as-synthesized PLGA. HepG2 cell membranes were obtained from the HepG2 cells through previous literatures 36 and applied as the cloak to functionalize the as-synthesized PLGA nanoparticles. As trypsin is one nonspecific proteolytic enzyme and is probable to destroy the activity of some proteins on the cell membrane, the formation of the cell membrane was characterized with some approaches. Transmission electron microscopy (TEM) was applied to characterize the morphologies and sizes of the bare PLGA and **HepM-PLGA** nanoparticles. The morphology and size of **HepM-PLGA** were like those of the bare PLGA (Figure 1A, 1B and Figure S1). The protein ingredient analysis of **HepM-PLGA** was verified with gel electrophoresis (Figure 1C), and the membrane protein profile of **HepM-PLGA** was like those of HepG2 cell membrane vesicles, illustrating that the membrane proteins within the HepG2 cell membrane were well retained during the biomimetic procedure. Since it has been reported that galectin-1, galectin-3 and CD47 are the main cellular adhesion molecules on cancer cell membranes that mediate the cell adhesion 41-43, the western blot (WB) analysis results (Figure 1D) illustrated the enrichment of galectin-1, galectin-3 and CD47 on the surface of **HepM-PLGA**. Conversely, as intracellular protein markers including histone H3 (a nuclear marker) and COXIV (a mitochondrial marker) were rarely found on the HepG2 cell membrane, the surface of** HepM-PLGA** was confirmed with the selective retention of membrane fragments (Figure 1D). The zeta-potential value of **HepM-PLGA** was determined to be -5.0 mV, close to that of the HepG2 cell membrane vesicles but quite different from that of the bare PLGA (Figure 1E). The slight gray shell outside **HepM-PLGA** in Figure 1B and the zeta-potential values shown in Figure 1E confirmed that the PLGA nanoparticles were successfully cloaked by the HepG2 cell membrane. The stabilities of **HepM-PLGA** and the bare PLGA nanoparticles were measured using a dynamic light scattering (DLS) analyzer (Figure 1F). After continuous measurement for 16 days, the bare PLGA nanoparticles grew obviously, while the size of **HepM-PLGA** showed little change, indicating the satisfactory stability of **HepM-PLGA**.

### Immunocompatibility assay

In drug delivery system, immunocompatibility usually plays a significant part. To verify the immunocompatibility of PLGA coated with carcinoma cell membrane, the immunocompatibility assay was carried out using RAW264.7 murine macrophage cell line and murine hepatocellular carcinoma cell membrane (H22 cells). In the assay, PLGA nanoparticles were coated with the H22 cell membranes (H22M-PLGA) and loaded with fluorescein isothiocyanate (FITC), and the bared PLGA nanoparticles with FITC were used as the control group. Macrophage cells were first incubated with FITC-H22M-PLGA and FITC-PLGA nanoparticles and then examined for particle internalization. After 4 h of incubation, the macrophage cells were washed and examined with confocal laser scanning microscopy (CLSM). As shown in Figure S2, the FITC-H22M-PLGA nanoparticles were less prone than the FITC-PLGA nanoparticles to be engulfed by macrophage cells, resulting in an approximately 75% reduction in particle internalization. Hence, it demonstrates that coating the PLGA nanoparticles with H22 cell membranes could effectively inhibit the murine macrophage cells uptake 39 and the carcinoma cell membrane coating could endow PLGA with excellent immunocompatibility.

### Validating the homologous targeting ability of HepM-PLGA

**Figure 2:**
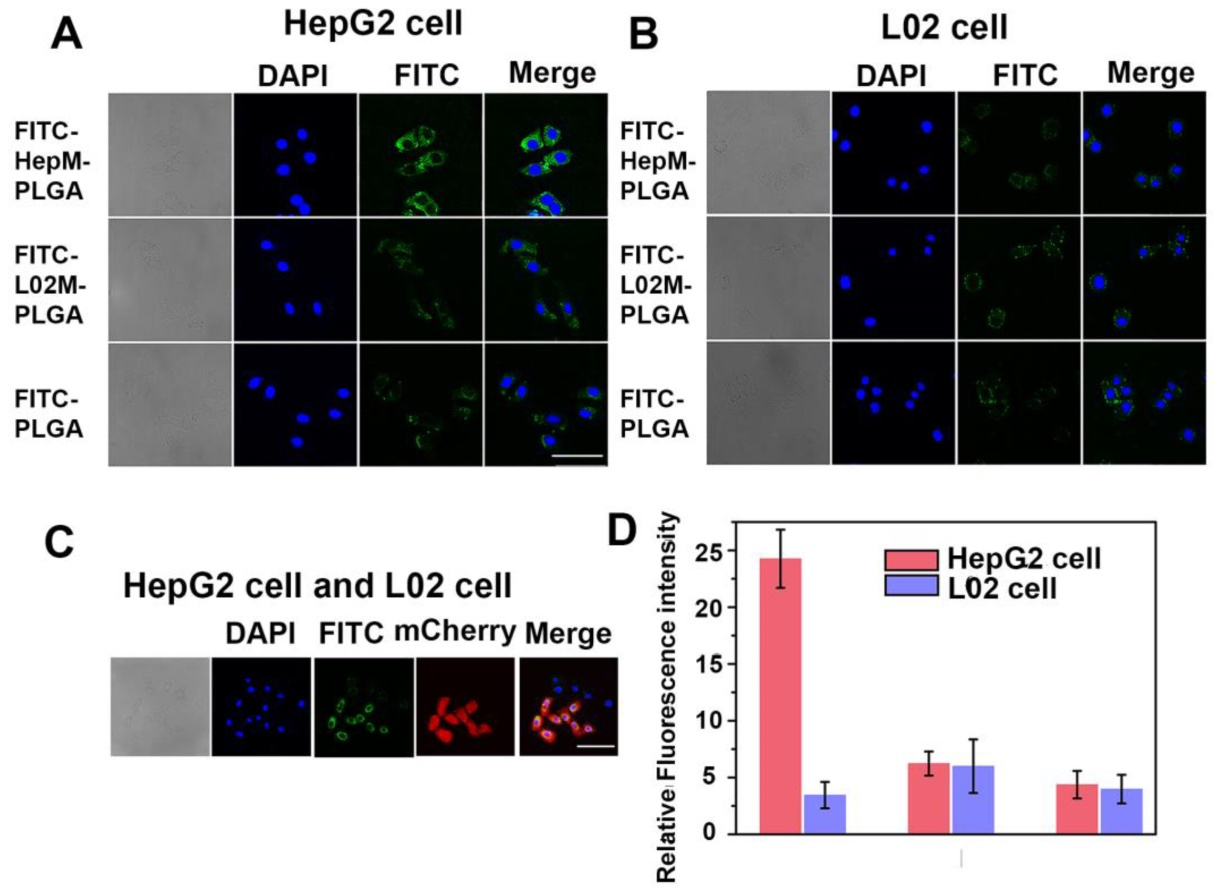
Validating the homologous targeting ability of **HepM-PLGA**. CLSM images of (A) the HepG2 cells and (B) the L02 cells after incubation with FITC-**HepM-PLGA**, FITC-L02M-PLGA and FITC-PLGA nanoparticles for 4 h. (C) CLSM images of the mixture of L02 cells and mCherry-labeled HepG2 cells upon co-incubation with FITC-**HepM-PLGA** nanoparticles for 4 h. (D) Quantitative histograms of the fluorescence intensities in (A) and (B). Scale bar: 75 μm.

To verify the homologous targeting ability of **HepM-PLGA** to HepG2 cells, HepG2 cells and L02 cells were incubated with FITC-**HepM-PLGA**, FITC- L02M-PLGA and FITC-PLGA nanoparticles and examined with CLSM and flow cytometry. As shown in Figure 2A & 2D, the fluorescence intensity of HepG2 cells treated with FITC-**HepM-PLGA** was approximately 4- to 5-fold stronger than that of the cells treated with the FITC-L02M-PLGA and FITC- PLGA nanoparticles, indicating that HepG2 cells could be recognized by **HepM-PLGA** *via* homologous aggregation. In contrast, there was little difference in the fluorescence intensities of the L02 cells incubated with FITC-**HepM-PLGA**, FITC-L02M- PLGA and FITC-PLGA (Figure 2B). Furthermore, as the mixture of L02 cells and transfected mCherry- labeled HepG2 cells incubated with FITC-**HepM- PLGA** for 4 h (Figure 2C), the stably transfected mCherry-labeled HepG2 cells showed red fluorescence, while L02 cells did not. The difference in the expression of red fluorescent protein distinguishes HepG2 from L02 cells obviously, demonstrating the homologous targeting ability of **HepM-PLGA**. The flow cytometric assay (Figure S3) further confirmed the homologous targeting ability of **HepM-PLGA** to HepG2 cells.

**Figure 3:**
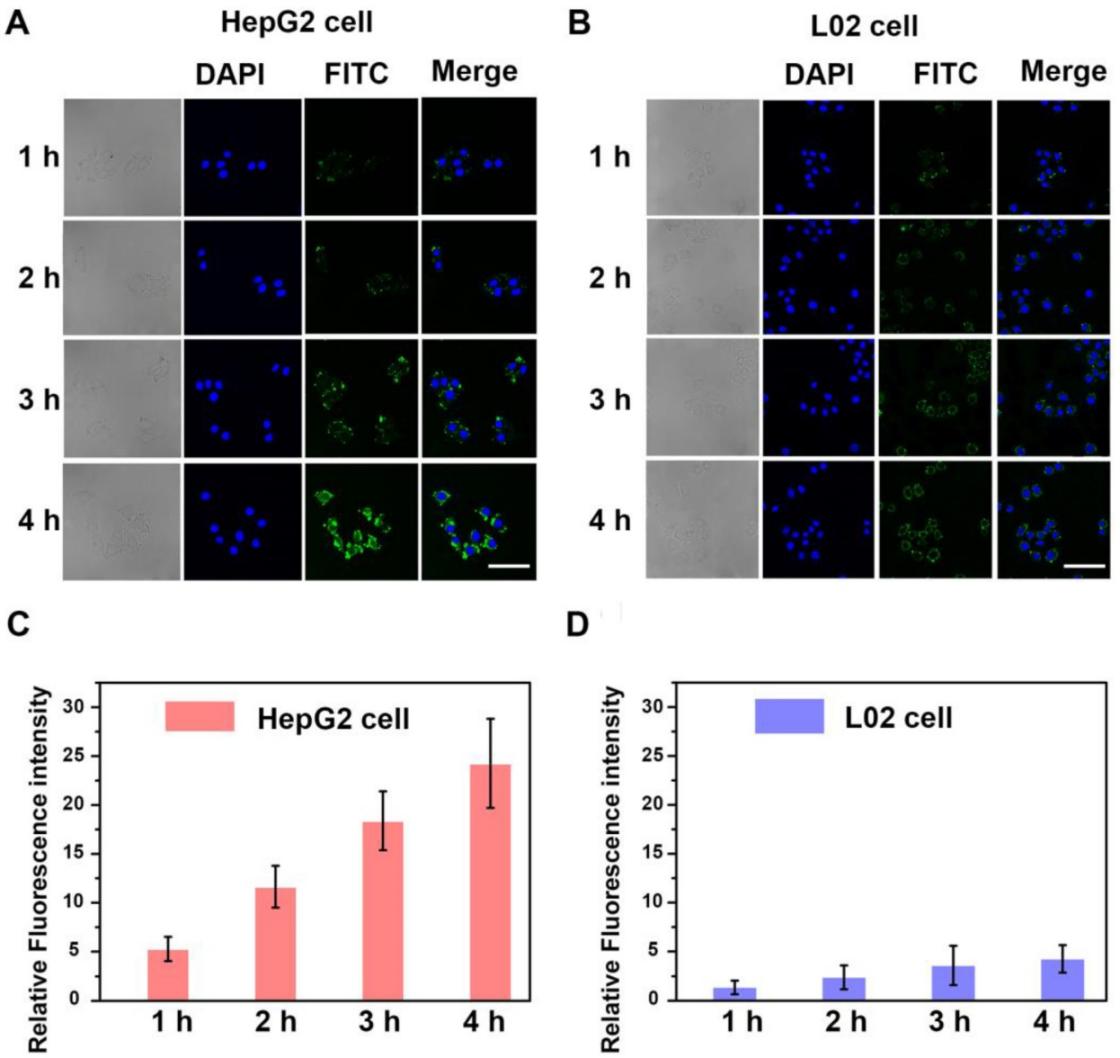
The time-dependent internalization of **HepM-PLGA**. CLSM images of (A) the HepG2 cells and (B) the L02 cells upon co-incubation with FITC-**HepM-PLGA** after 1, 2, 3 and 4 h. (C) & (D) Quantitative histograms of fluorescence intensities for (A) & (B), respectively. Scale bar: 75 μm.

**Figure 4:**
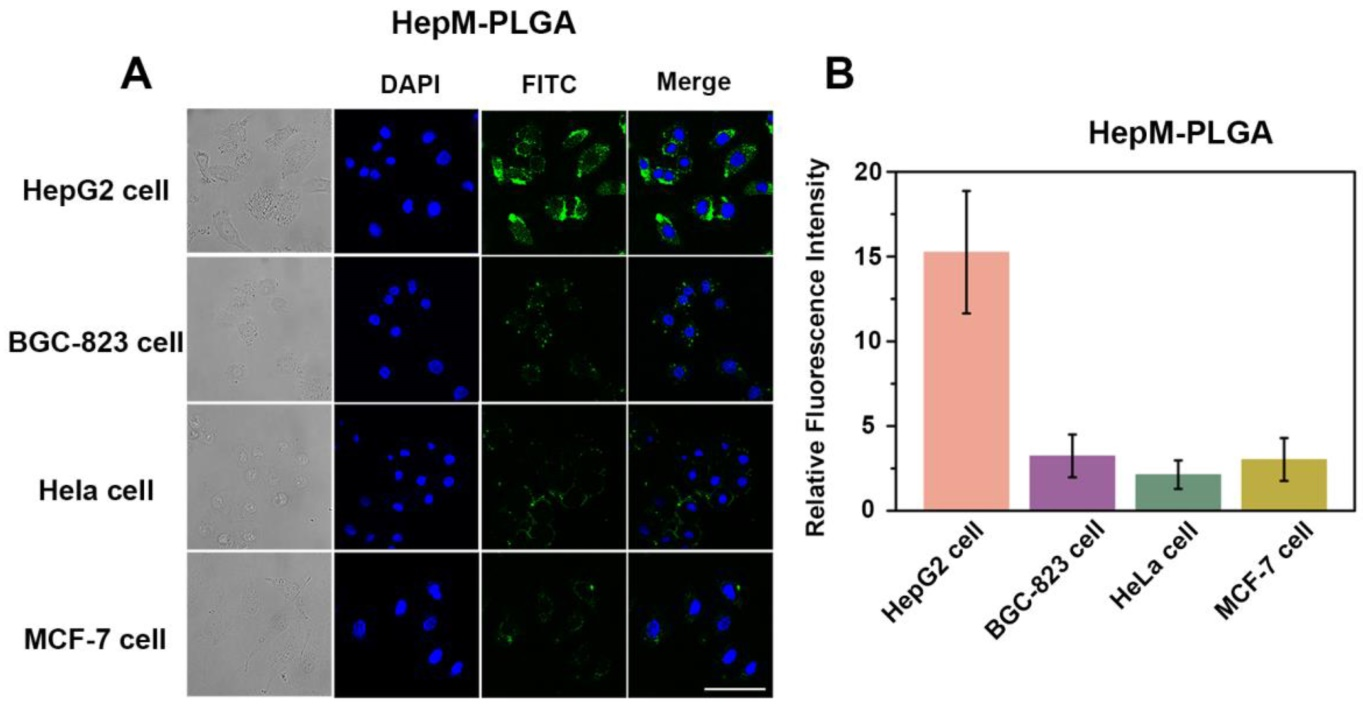
The targeting ability of **HepM-PLGA** to HepG2 cells and other cancer cells. (A) CLSM images of HepG2 cells, BGC-823 cells, HeLa cells and MCF-7 cells incubated with FITC-**HepM-PLGA** nanoparticles. (B) Quantitative histogram of the fluorescence intensities for the images in (A). Scale bar: 75 μm.

The homotypic-mediated internalization pathway of **HepM-PLGA** by HepG2 cells was further investigated (Figure 3). The fluorescence intensity enhances with time, indicating the time-dependent internalization pathway of **HepM-PLGA** by HepG2 cells. To further verify the homotypic aggregation of cancer cells and the homologous targeting ability of **HepM-PLGA**, certain kinds of cancer cells, including human gastric carcinoma cells (BGC-823 cells), cervical carcinoma cells (HeLa cells) and breast cancer cells (MCF-7 cells), were incubated with FITC-**HepM-PLGA**. The CLSM images and the flow cytometric assay exhibit FITC-**HepM-PLGA** with a unique homologous targeting ability to HepG2 cells rather than other kinds of cancer cells (Figure 4 & S4). Similarly, the FITC-MCFM-PLGA nanoparticles could only recognize MCF-7 cells rather than other cancer cells (Figure S5 & S6). Besides, in consideration of other cell membranes including red blood cell (RBC), white blood cell (WBC) and platelet (PLT) were used for nanoparticle functionalization as well 44-46, the unique capabilities of FITC-**HepM-PLGA** was compared with PLGA nanoparticles coated by RBC membranes (FITC-RBCM-PLGA). The FITC-RBCM- PLGA nanoparticles were incapable of targeting tumor cells specifically (Figure S7). Taken together, the **HepM-PLGA** nanoparticles exclusively target HepG2 cells *via* the homologous aggregation effect, and such homologous targeting laid the foundation for the targeted chemotherapy of HCC.

### *In vitro* therapeutic effect

**Figure 5:**
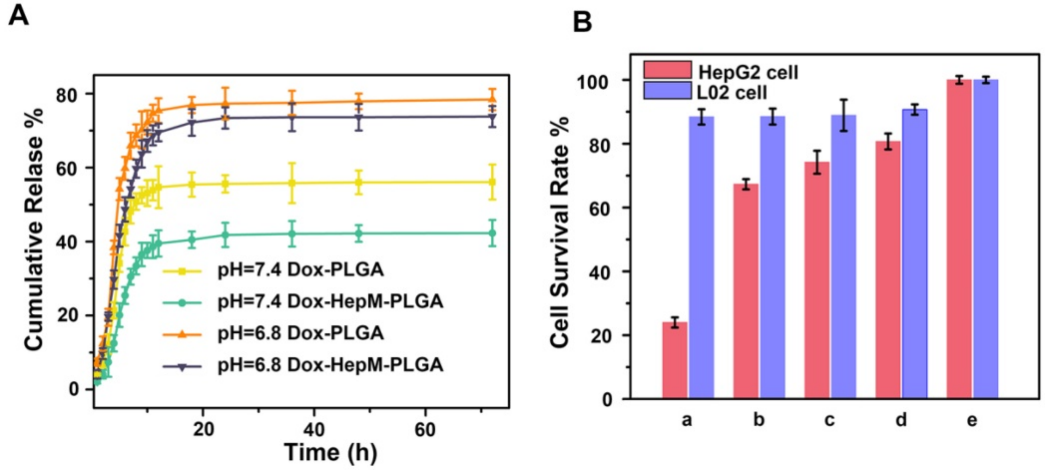
Drug release and MTT assay. (A) The *in vitro* release profiles of Dox-**HepM-PLGA** and Dox-PLGA at pH 7.4 and 6.8. (B) MTT assay results. The concentration of Dox was 5 μg/mL, a-e refer to Dox-**HepM-PLGA**, Dox-L02M-PLGA, Dox-PLGA, free Dox, and PBS, respectively.

The carrying and therapeutic efficiency of **HepM-PLGA** as drug carrier platform are curial for the clinic application 38, 47, 48. Herein, Dox was chosen as the model anticancer drug, and the loading content was determined to be 38.88 μg/mg (Figure S8). Afterwards, the *in vitro* release profiles of Dox loaded into **HepM-PLGA** and bare PLGA were investigated (Figure 5A). At pH 7.4 (mimicking the normal cell microenvironment), Dox-PLGA displayed a fast release of more than 55% of the drug during the initial 10 h, but only less than 38% for the Dox-**HepM-PLGA** group, then followed by a stage with slower release. Compared to Dox-PLGA, Dox-**HepM-PLGA** possesses a lower drug release rate, thereby inhibiting the release of the drug Dox in blood circulation to some extent. At pH 6.8 (mimicking the tumor cell microenvironment) 49, a burst release of approximately 70% Dox occurred during the first 10 h, and followed by a continuous release, suggesting its acid-responsive behavior. Hence, the **HepM-PLGA** drug nanocarrier platform is suitable for application in the slightly acidic tumor microenvironment.

**Figure 6:**
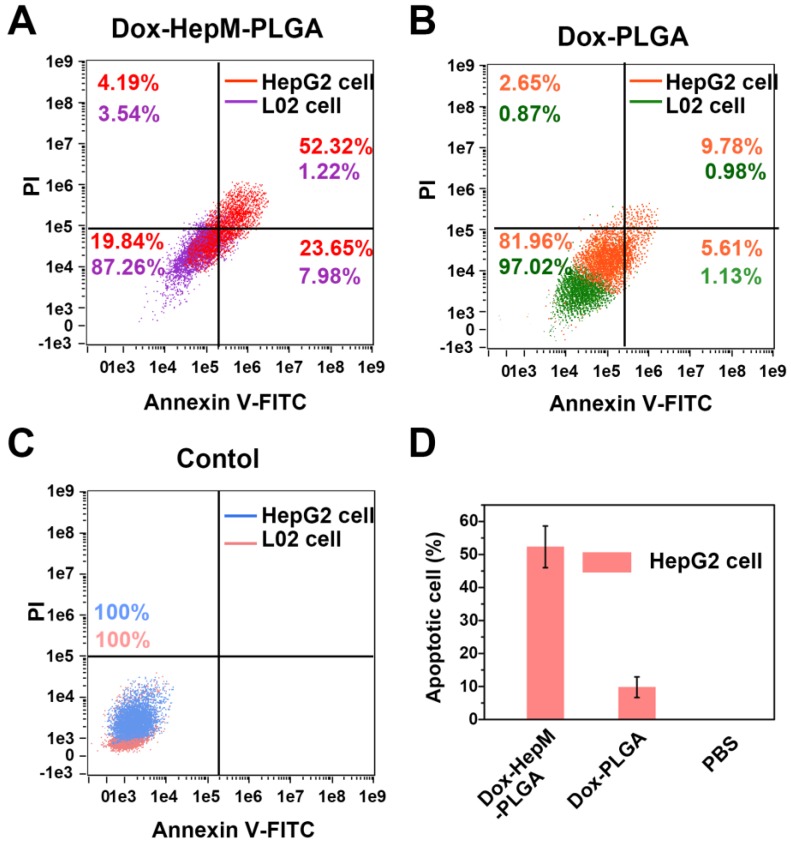
(A) to (C) Flow cytometry results of HepG2 cells and L02 cells after incubation with Dox-**HepM-PLGA**, Dox-PLGA and PBS for 4 h and being stained with an Annex V-FITC/PI apoptosis kit, respectively. (D Apoptotic rate of HepG2 cells: corresponding to (A) to (C). Dox concentration: 5 μg/mg.

For the next step, the *in vitro* therapeutic effect of Dox-**HepM-PLGA** was evaluated. First, the MTT assay results illustrate the therapeutic efficacy and targeting ability of Dox-**HepM-PLGA** on the HepG2 cells (Figure 5B & S9). With the concentration of Dox of 5 μg/mL, the HepG2 cell viability of the Dox-**HepM-PLGA** group was as low as 24%, while the viability of L02 cells treated with Dox-**HepM- PLGA** was up to 87%. Other groups of cells treated with Dox-L02M-PLGA, Dox-PLGA and free Dox showed comparable viabilities. Furthermore, to study the apoptosis of HepG2 cells treated with Dox-**HepM- PLGA**, HepG2 cells and L02 cells were respectively incubated with Dox-**HepM-PLGA**, Dox-PLGA, and PBS for 4 h, and stained with Annexin V-FITC/PI, then analyzed with flow cytometry. Considering the absorbance and emission peaks of DOX overlapping significantly with PI and FITC, HepG2 cells were separately incubated with Dox-**HepM-PLG** for 4 h and analyzed with flow cytometry, the collected fluorescence signal was compared with that collected after HepG2 cells were stained with Annexin V-FITC/PI (Figure S10A). Fluorescence signal from DOX was negligible and showed little interference with the fluorescence signal from FITC. In fact, it is known that PI and FITC interfere with each other in the apoptosis assay, therefore, the data was processed with compensation matrix (Figure 6). Compared to the control groups (Figure S10B), Dox-**HepM-PLGA** strongly induced apoptosis of the HepG2 cells. After 4 h of incubation with Dox-**HepM-PLGA**, about half of the HepG2 cells were in the late apoptotic stage (Figure 6A, upper right quadrant, annexin V+/PI+), while in other groups the percentages of the late apoptotic HepG2 cells or L02 cells were significantly lower. Overall, Dox-**HepM-PLGA** possesses an excellent *in vitro* therapeutic effect on HepG2 cells, which makes it a perspective applicant for *in vivo* antitumor chemotherapy.

### *In vivo* tumor image and antitumor effect

**Figure 7:**
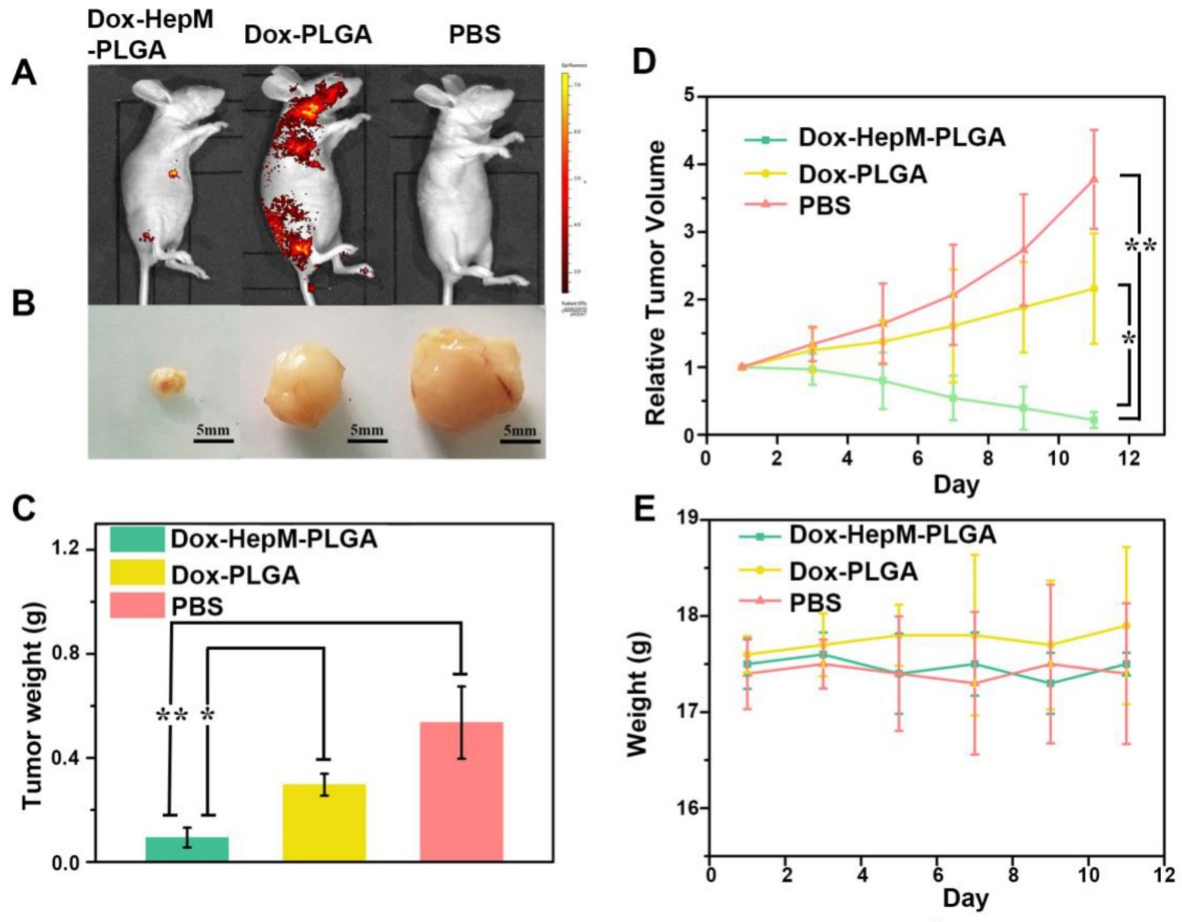
*In vivo* tumor imaging and antitumor effect. (A) Fluorescence image of HepG2 tumor-bearing nude mice 11 days after the intravenous injection of Dox-**HepM-PLGA** and its counterparts. (B) Photos of the tumors extracted from the nude mice bearing the HepG2 tumor 11 days after the intravenous injection of Dox-**HepM-PLGA** and its counterparts. (C) Weights of the tumors extracted from the nude mice in (B). (D) Quantitative results of the HepG2 tumor relative volumes during chemotherapy. (E) Body weights of the nude mice during chemotherapy. All bars represent means±s.d. *P≤0.05, **P≤0.01.

The satisfactory *in vitro* therapeutic results inspired us to evaluate the *in vivo* antitumor effects. The accumulation of **HepM-PLGA** loaded with Dox in the nude mice bearing a HepG2 tumor was investigated by fluorescence imaging of Dox 11 days after the intravenous injection. The fluorescence signal in the tumor regions of the **HepM-PLGA** group was the strongest, while signals in the other groups were less intensified (Figure 7A). Specifically, the Dox accumulation in the tumor was dramatically enhanced in the **HepM-PLGA** group and the drug delivery efficiency of **HepM-PLGA** was relatively high, which is beneficial for improving the *in vivo* antitumor efficacy. The visual images of the extracted tumors illustrate the suppression effect of Dox- **HepM-PLGA** on the tumor, which was also observed in the tumor weight histograms (Figure 7B & 7C). Moreover, the tumor volume in the nude mice treated with Dox-**HepM-PLGA** decreased approximately 90%, while a two-fold increase was observed in the Dox-PLGA group (Figure 7D). In Figure 7E, the profile verifies no obvious change in the body weights of the treated nude mice in all the groups. These results combined with Figure S11 and S12 in the SI confirmed that the homologous targeting ability of **HepM-PLGA** accounted for the accumulation in the tumor region and the therapeutic effect of Dox.

**Figure 8:**
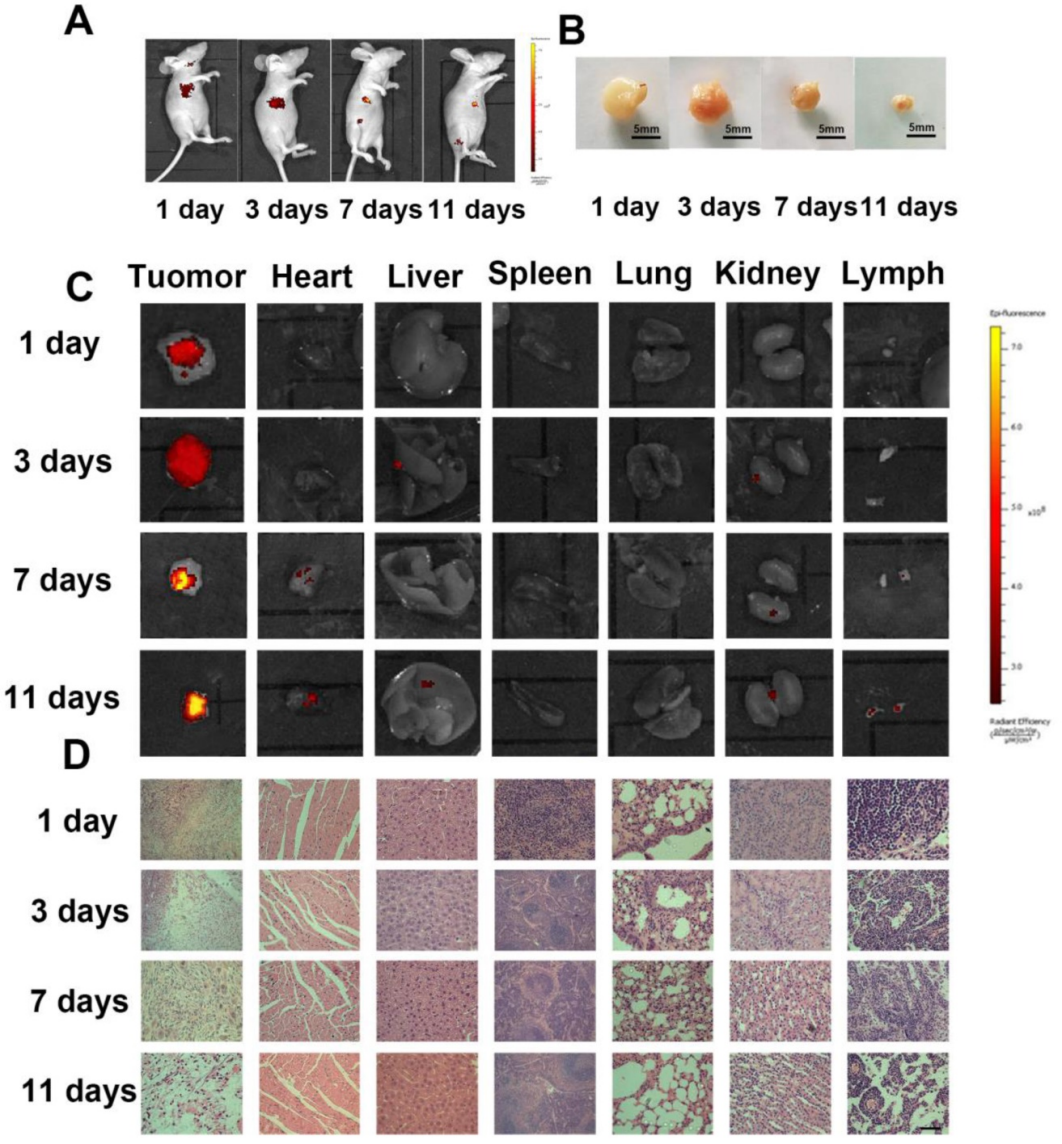
*In vivo* tumor image and antitumor effect of the Dox**-HepM-PLGA**. (A) Fluorescence image of HepG2 tumor-bearing nude mice 3 days,7 days and 11 days after the intravenous injection of Dox-**HepM-PLGA**. (B) Photos of *ex vitro* the tumors extracted from the nude mice bearing the HepG2 tumor 3 days, 7 days and 11 days after the intravenous injection of Dox-**HepM-PLGA**. (C) *Ex vitro* fluorescence images of the major organs and tumors tissues extracted from the nude mice bearing the HepG2 tumor 3 days, 7 days and 11 days after the intravenous injection of Dox-**HepM-PLGA** and its counterparts. (D) Hematoxylin and Eosin (H&E) staining of the tissue slices of HepG2 tumor-bearing nude mice 3 days,7 days and 11 days after the intravenous injection of Dox-**HepM-PLGA**. Scale bar: 50 μm.

The time-dependent accumulation and distribution of Dox-**HepM-PLGA** in the HepG2 tumor- bearing nude mice and histological analysis of the *ex vitro* organs were explored. The fluorescence intensity in the mice's tumor region increased with treatment time while the relative tumor volume decreased dramatically, while the strongest Dox signal in the tumor region was observed in the 11 days panel (Figure 8A-C). Additionally, in the mice subjected to Dox-PLGA group, the Dox signal in the heart, liver, kidney, lung and lymph were significant enhanced, and there was apparent liver injury and congestion of the alveolar walls. In comparison, in the mice subjected to Dox-**HepM-PLGA** group, only weak Dox signal and barely tissue damage were observed in all the extracted organs (Figure 8D & S13). It was indicated **HepM-PLGA** was characterized of low toxicity and decent biocompatibility.

## Conclusions

In summary, to improve the low targeting ability, efficiency and immunocompatibility of anticancer drug nanocarrier systems, we took full advantage of a homotypic cancer cell membrane and designed a novel drug nanocarrier platform **HepM- PLGA** *via* a biomimetic strategy for HCC chemotherapy. In **HepM-PLGA**, the PLGA core was cloaked by HepG2 cell membranes that exhibit a unique homotypic aggregation effect. **HepM-PLGA** was characterized to own high stability, great immunocompatibility and excellent homotypic targeting ability toward HepG2 cells. *In vivo* experiment verifies Dox-**HepM-PLGA**'s excellent therapeutic effect on the tumor of the nude mice, while the damage to the major organs was negligible. **HepM-PLGA** is believed to serve as promising and robust nanoplatform for HCC chemotherapy and provide a new strategy for the design of an ideal drug delivery platform for other cancers.

## Experimental Section

### Preparation of PLGA nanoparticles

PLGA (Mw = 30,000, 50 mg) was first dissolved in dichloromethane (2 mL) and then mixed with secondary water (200 μL), followed by sonication for 5 min (350 W) to form the first emulsion. This first emulsion was mixed with 8 mL of 1% PVA solution under sonication for another 15 min, forming the multiple emulsion. The multiple emulsion was then added to 80 mL of 2% isopropyl alcohol solution slowly and stirred overnight. The supernatant was collected after centrifugation treatment at 3,000 rpm for 10 min, and then, the PLGA nanoparticles were collected by centrifugation treatment at 14,000 rpm for 15 min and washed three times. Finally, the PLGA nanoparticles were resuspended in 5 mL of secondary water and lyophilized for 20 h. Then, the synthesized PLGA nanoparticles were dissolved in methylene chloride and mixed with the FITC stock solution (DMSO as the solvent) to load FITC. When the synthesized PLGA nanoparticles were used to load Dox, Dox was directly dissolved in the internal aqueous phase during the PLGA preparation.

### Preparation of HCC cell membranes

Human HCC cell HepG2 cell were seeded in cell culture dishes and incubated with Dulbecco's modified Eagle's medium (DMEM) containing 10% FBS and 1% antibiotics (penicillin-streptomycin). After the HepG2 cells were covered, the cells were detached with trypsin, isolated by centrifugation at 1,000 rpm for 2 min, and then washed with PBS. The collected cells were resuspended in 800 μL of RIPA lysate containing 1% phenylmethanesulfonyl fluoride (PMSF) for 30 min at 4 °C. Then, the supernatant solution was collected after centrifugation at 12,000 rpm for 8 min at 4 °C. To collect the cell membrane vesicles, the supernatant was subjected to further centrifugation at 20000 rpm for 60 min. The cell membrane vesicles were resuspended in PBS and saved at -80 °C.

### Preparation of human normal liver cell membrane

Human normal liver cell L02 cell line were used. The preparation procedure for L02 cell membrane was the same as the preparation process for HepG2 cell membranes.

### Preparation of cell membrane cloaked PLGA nanoparticles

Two milliliters of PLGA nanoparticles (1.0 mg/ mL) was mixed with 1 mL of HepG2 cell or L02 cell membrane vesicles (0.5 mg/mL). Then, the mixture was sonicated for 15 min (40 kW).

### Immunocompatibility assay of HepM-PLGA

Cells of the RAW264.7 macrophage cell line were seeded in a confocal cell culture dish and cultured for 24 h in 2 mL of DMEM with 10% FBS. After the supernatant was discarded, the RAW264.7 cells were incubated with FITC-**HepM-PLGA**, FITC-L02M- PLGA and FITC-PLGA. To prepare the incubation buffer, 100 μL of FITC-**HepM-PLGA**, FITC-L02M- PLGA and FITC-PLGA was mixed with 900 μL of DMEM containing 10% FBS. The RAW264.7 cells were incubated with 200 μL of the incubation buffer in every well for 4 h. The incubation buffer was discarded, and the cells were washed three times with PBS (pH 7.4). Then, the cells were imaged immediately using a confocal microscope with an objective lens (× 63). Excitation of the probe-treated cells at 488 nm was performed using an argon laser, and the emitted light was collected with a META detector between 520 and 550 nm. The relative fluorescence intensities were measured by Zen software.

### Validating the homologous targeting property of HepM-PLGA with confocal fluorescence imaging

HepG2 cells or L02 cells were seeded in a confocal cell culture dish and cultured for 24 h in 2 mL of DMEM with 10% FBS. After the supernatant was discarded, HepG2 cells and L02 cells were incubated with FITC-**HepM-PLGA**, FITC-L02M-PLGA and FITC-PLGA, respectively. To prepare the incubation buffer, 100 μL of FITC-**HepM-PLGA**, FITC-L02M- PLGA and FITC-PLGA was mixed with 900 μL of DMEM containing 10% FBS. HepG2 cells and L02 cells were incubated with 200 μL of the incubation buffer for in every well 4 h. The incubation buffer was discarded, and the cells were washed three times with PBS (pH 7.4). Then, the cells were imaged immediately using a confocal microscope with an objective lens (× 63). Excitation of the probe-treated cells at 488 nm was performed using an argon laser, and the emitted light was collected with a META detector between 520 and 550 nm. The relative fluorescence intensity was measured by Zen software.

### *In vitro* drug release

The Dox-**HepM-PLGA** nanoparticles and Dox-PLGA nanoparticles drug release concentration *in vitro* at different pH conditions was measured by the standard curve method. Dox-**HepM-PLGA** nanoparticles and Dox-PLGA nanoparticles were placed in a dialysis tube, and dialysis tubing closures were used to close both ends of the opening. The dialysis tubes were placed in PBS (37 °C) solution at pH 7.4 and pH 6.8. At several times, 2 mL of PBS solution was removed for concentration determination, and the samples were supplemented with 2 mL of PBS. Then, the Dox-PBS standard solutions were examined. The absorption of Dox was measured at the 480 nm wavelength by a UV-visible-NIR spectrophotometer (HITACHI, Japan), and the standard curve was plotted. According to the standard curve method, the PLGA particle drug release was measured.

### MTT analysis

HepG2 cells and L02 cells were inoculated into sterile 96-well plates, and 200 μL of DMEM containing 10% FBS was added to each well (excluding the outermost well) for 24 h, and the culture solution was discarded. Configured incubation solution: Dox- **HepM-PLGA** solution, Dox-L02M-PLGA solution, Dox-PLGA, and the same concentration of free Dox drug were mixed with DMEM containing 10% FBS as an incubation solution. L02 cells and HepG2 cells were incubated with the final concentration of Dox at 5, 3 and 1 μg/mL for 4 h. The incubation solution was then discarded and washed three times with PBS, added to a 96-well plate with DMEM containing 10% FBS, and 20 μL of MTT (5 mg/mL) was added to each well, and incubated for 4 hours. The medium was then discarded and 100 μL of DMSO was added. After 20 minutes of shock. The absorbance at 490 nm was measured with a microplate reader.

### The *in vitro* therapeutic effect of Dox-HepM-PLGA assessed with flow cytometry

HepG2 cells or L02 cells were seeded in a confocal cell culture dish and cultured for 24 h in 2 mL of DMEM with 10% FBS. After the supernatant was discarded, the HepG2 cells and L02 cells were incubated with Dox-**HepM-PLGA**, Dox-PLGA and PBS. To prepare the incubation buffer, 100 μL of Dox-**HepM-PLGA**, Dox-PLGA or PBS was mixed with 900 μL of DMEM containing 10% FBS. The HepG2 cells and L02 cells were incubated with 200 μL of the incubation buffer for in every well for 4 h. After the incubation buffer was discarded, the cells were trypsinized (free from EDTA), collected by centrifugation at 1000 rpm for 2 min and washed thrice with PBS. Finally, the cells were stained by the Annexin V-FITC/PI Apoptosis Detection Kit and examined by flow cytometry. All flow cytometry studies were conducted on an Image-StreamX multispectral imaging flow cytometer (Amnis Corporation), and the data were analyzed using IDEAS software.

### *In vivo* tumor image

Select 4 to 6 weeks of BALB/c nude mice weighing 15-20 g were used. The mice were housed in cages (5 per cage) and regularly fed rat chow and water. To build a solid tumor of liver cancer in nude mice subcutaneously, 3 × 10^6^ HepG2 cells were injected subcutaneously into the flank region of the nude mice. Tumor volume = (major diameter of tumor) × (minor diameter of tumor)^2^/2.

When the tumor volume of the nude mice reached 100-200 mm^3^, the mice were randomly divided into 3 groups and intravenously injected with Dox-**HepM-PLGA** or the counterparts every other day. The DOX dose was 2.5 mg/kg per mouse in every group. Group 1 was intravenously injected with 100 μL of PBS and was the control group, Group 2 was intravenously injected with Dox-PLGA solution, Group 3 was intravenously injected with Dox-**HepM- PLGA**.

To verify the effect of drug treatment, the above groups of mice were treated for 3 days, 7 days and 11 days and imaged by the *in vivo* imaging system. Then, 24 h after the final injection, the nude mice were sacrificed, and the main organs (heart, liver, spleen, lung, kidney and lymph) and tumors were extracted for *ex vitro* imaging. All animal experiments were carried out according to the Principles of Laboratory Animal Care (People's Republic of China) and the Guidelines of the Animal Investigation Committee, Biology Institute of Shandong Academy of Science, China. The statistical data were analyzed using SPSS Statistics software, for deriving standard deviation, one-way ANOVA test and Bonferroni test. A p-value of 0.05 was taken as the level of significance and the data were labeled with (*) for P < 0.05, and for (**) for P < 0.01, Each experiment was conducted in triplicate (n=3).

## Supplementary Material

Supplementary figures and tables.

## Abbreviations

PLGA: poly (lactic-co-glycolic acid)
Dox: Doxorubicin
HepM: homotypic HepG2 cell membrane
HepM-PLGA: homotypic HepG2 cell membrane- cloaked PLGA nanocarrier
TEM: transmission electronic microscopy
