# Supplementary material for: Homotypic Cell Membrane-Cloaked Biomimetic Nanocarrier for the Targeted Chemotherapy of Hepatocellular Carcinoma
Source: Theranostics. 2019 Aug 12;9(20):5828–38. doi: 10.7150/thno.34837 (PMC6735366; doi:10.7150/thno.34837)
Supplement: Supplementary file 1 — Supplementary figures and tables. [file thnov09p5828s1.pdf]

Supporting Information

Homotypic cell membrane-cloaked biomimetic nanocarrier for the targeted chemotherapy of hepatocellular carcinoma

Xiaojun Liu†, Yingxue Sun†, Shushen Xu, Xiaonan Gao, Fanpeng Kong, Kehua Xu*, and Bo Tang*

College of Chemistry, Chemical Engineering and Materials Science, Key Laboratory of Molecular and Nano Probes, Ministry of Education, Collaborative Innovation Center of Functionalized Probes for Chemical Imaging in Universities of Shandong, Institute of Molecular and Nano Science, Shandong Normal University, Jinan 250014, P. R. China.

*Corresponding author. E-mail: tangb@sdnu.edu.cn (B. Tang), xukehua@sdnu.edu.cn (K. Xu)

Tel: 86-531-8618-2544; Fax: 86-531-8618-6033 (8618-0304)

All authors have given approval to the final version of the manuscript.

†These authors contributed equally.

Contents:

1. Materials and instruments
2. Experimental section
3. TEM image of PLGA nanoparticles and HepG2 cell membrane
4. Immunocompatibility assay of H22M-PLGA
5. Flow cytometry about the homologous targeting property of FITC-HepM-PLGA
6. The targeting ability of **HepM-PLGA** toward HepG2 cells and other cancer cells
7. The targeting ability of MCFM-PLGA toward MCF-7 cells and other cancer cells
8. The targeting ability of RBCM-PLGA
9. Dox loading content standard curve
10. MTT assay
11. In vitro therapeutic effect
12. In vivo tumor image and antitumor effect after 3 days
13. In vivo tumor image and antitumor effect after 7 days
14. Fluorescence images of the major organs and tumors tissues

1. Materials and instruments

PLGA (50:50, Mw=30000) and PVA (polyvinyl alcohol, Mw=10,000 to 26,000), 86%-89% mole hydrolyzed were purchased from Sigma-Aldrich. MTT (3-(4,5-dimethyl-2-thiazolyl)-2,5-diphenyl-2-H-tetrazolium bromide) and Fluorescein isothiocyanate (FITC) was purchased from Shanghai Aladdin Biochemical Technology Co., Ltd. Isopropyl alcohol and DMSO were purchased from Sinopharm Chemical Reagent Co., Ltd. Solvents and regents were all of analytical grade and purchased from China National Pharmaceutical Group Corp. (Shanghai, China). All solutions were prepared using ultrapure water (18.2 MΩ·cm, Millipore). The pH buffer buffer (PBS, pH 3−9) were prepared using 0.1 M citric acid and 0.2 M disodium hydrogen phosphate adjusted with 2 M NaOH or HCl solutions. The human hepatocellular liver carcinoma cell line, HepG2, normal liver cell line L02, human gastric carcinoma cells (BGC-823 cells), cervical carcinoma cells (HeLa cells) and breast cancer cells (MCF-7 cells) were obtained from the Committee on Type Culture Collection of the Chinese Academy of Sciences. RIPA lysate was obtained from Beyotime Institute of Biotechnology. Annexin V-FITC/PI was purchased from Beijing jiamei new biological technology Co., Ltd.

Transmission electron microscopy (TEM) characterization were taken on a JEOL transmission electron microscope (JEM-2100, Shimadzu, Japan) operated at 200 kV. UV-vis absorption spectra were measured on a UV-visible spectrophotometer (UV-1700 Pharmaspec, Shimadzu, Japan). Dynamic light scattering (DLS) and zeta potential measurements were carried out on Malvern Zeta Sizer Nano (Malvern Instruments). Gel electrophoresis analysis and western bloot analysis were imaging by ChemiDocTM Touch Imaging system (Bio-Rad, Hercules, CA, USA). All pH measurements were performed with a pH-3c digital pH-meter (Shanghai LeiCi Device Works, Shanghai, China) with a combined glass-calomel electrode. Confocal fluorescence imaging was performed with a TCS SP8 confocal laser scanning microscopy (CLSM, Leica Co., Ltd. Germany) with an objective lens (×63). In vivo fluorescence imaging was conducted on the in vivo imaging system (IVIS, PerkinElmer). Absorbance was measured with the microplate reader (RT 6000, Rayto, USA) in the MTT assay. The flow cytometry data were obtained using image-Stream X multispectral imaging flow cytometer (Amnis Corporation).

2. Experimental section

2.1 TEM characterization.

Negative staining (1% phosphotungstic acid) for TEM was used to characterize the bare PLGA nanoparticles and the cell membrane-cloaked PLGA nanoparticles and HepG2 cell membrane. The nanoparticles were dropped on a copper grid for 5 min, and then another sample was negatively stained with 2 drops of 1 wt% phosphotungstic acid. After the solution was completely dry, TEM characterization was carried out on a JEM-2100.

2.2 Gel electrophoresis analysis and western blot analysis.

HepG2 cell lysates and HepG2 cell membrane protein extraction solution was added to SDS loading buffer in a 4:1 ratio and then heated at 95-100 °C for 5 min for protein denaturation. Twenty microliters of protein sample and protein marker were sequentially and slowly added to the loading wells of an SDS-PAGE gel containing 12% separation gel and 5% spacer gel, the voltage was then set to 80 V for electrophoresis in the electrophoresis apparatus, and finally the voltage was changed to 120 V until the sample reached the spacer gel. The proteins in the resulting polyacrylamide gel were stained with Coomassie brilliant blue stain. The gel was imaged by the ChemiDoc^{TM} Touch Imaging System (Bio-Rad, Hercules, CA, USA).

The proteins in the resulting polyacrylamide gel were then transferred to a PVDF membrane (PerkinElmer) at 100 V for 60 min, while the Trans-Blot system was placed in ice water. The obtained PVDF membrane was blocked with 5% nonfat milk powder at room temperature for 1 h. Then, the PVDF membranes were incubated with primary antibodies overnight at 4°C. The primary antibodies included antibodies to galectin-1, galectin-3, CD47, cytochrome c oxidase (cytoplasmic markers), and histone H3 (nuclear marker). The next day, the rewarmed PVDF membrane was washed with TBST three times, incubated with goat anti-mouse lgG HRP conjugate (1:5000 dilution in 5% nonfat milk powder) for 1 h, and then washed thrice with TBST. Finally, ECL chemiluminescent coloring solution was added dropwise, and the PVDF membranes were imaged by ChemiDoc^{TM} Touch Imaging system (Bio-Rad, Hercules, CA, USA).

2.3 Validating the homologous targeting property of HepM-PLGA with confocal fluorescence imaging.

HepG2 cells, BGC-823 cells, HeLa cells and MCF-7 cells were seeded in a confocal cell culture dish and cultured for 24 h in 2 mL of DMEM with 10% FBS. After the supernatant was discarded,

the HepG2 cells, BGC-823 cells, HeLa cells and MCF-7 cells were incubated with FITC-**HepM-PLGA**. To prepare the incubation buffer, 100 μL of FITC-**HepM-PLGA** was mixed with 900 μL of DMEM containing 10% FBS. The HepG2 cells, BGC-823 cells, HeLa cells and MCF-7 cells were incubated with 200 μL of the incubation buffer in every well for 4 h. The incubation buffer was discarded, and the cells were washed three times with PBS (pH 7.4). Then, the cells were imaged immediately using a confocal microscope with an objective lens (× 63). Excitation of the probe-treated cells at 488 nm was performed using an argon laser, and the emitted light was collected with a META detector between 520 and 550 nm.

2.4 Generation of stably transfected cell lines

The HepG2 cells were seeded onto a 24-well plate with 8×104 cells peer well. After 12 hours, the cells are completely attached to the wall. Replace the original culture medium with fresh culture medium and add 10 μL mCherry-loaded-lentiviral vector suspension. After mixing, the culture was continued for 24 hours. The culture medium containing virus was replaced with fresh medium to culture the cells. Stable transfected mCherry-HepG2 cell lines were obtained by purinomycin (1.0 μg /mL) screening 72 h after transfection.

2.5 The time-dependent internalization of HepM-PLGA by HepG2 cells

HepG2 cells or L02 cells were seeded in a confocal cell culture dish and cultured for 24 h in 2 mL of DMEM with 10% FBS. After the supernatant was discarded, HepG2 cells or L02 cells were incubated with FITC-**HepM-PLGA** for 1 h, 2 h, 3 h or 4 h. To prepare the incubation buffer, 100 μL of **FITC-HepM-PLGA** was mixed with 900 μL of DMEM containing 10% FBS. HepG2 cells or L02 cells were incubated with 200 μL of the incubation buffer for in every well. The incubation buffer was discarded, and the cells were washed three times with PBS (pH 7.4). Then, the cells were imaged immediately using a confocal microscope with an objective lens (× 63). Excited at 488 nm using an argon laser, and the emitted light was collected with a META detector between 520 and 550 nm. The relative fluorescence intensity was measured by Zen software.

2.4 Validating the homologous targeting property of MCFM-PLGA with confocal fluorescence imaging.

HepG2 cells, BGC-823 cells, HeLa cells and MCF-7 cells were seeded in a confocal cell culture dish and cultured for 24 h in 2 mL of DMEM with 10% FBS. After the supernatant was discarded,

the HepG2 cells, BGC-823 cells, HeLa cells and MCF-7 cells were incubated with FITC-MCFM-PLGA. To prepare the incubation buffer, 100 μL of FITC-MCFM-PLGA was mixed with 900 μL of DMEM containing 10% FBS. HepG2 cells, BGC-823 cells, HeLa cells and MCF-7 cells were incubated with 200 μL of the incubation buffer for in every well for 4 h. The incubation buffer was discarded, and the cells were washed three times with PBS (pH 7.4). Then, the cells were imaged immediately using a confocal microscope with an objective lens (× 63). Excitation of the probe-treated cells at 488 nm was performed using an argon laser, and the emitted light was collected with a META detector between 520 and 550 nm. The relative fluorescence intensity was measured by Zen software.

2.5 Validating the homologous targeting property of HepM-PLGA with flow cytometry.

HepG2 cells, BGC-823 cells, HeLa cells and MCF-7 cells were seeded in a confocal cell culture dish and cultured for 24 h in 2 mL of DMEM with 10% FBS. After the supernatant was discarded, the HepG2 cells, BGC-823 cells, HeLa cells and MCF-7 cells were incubated with FITC-**HepM-PLGA**. To prepare the incubation buffer, 100 μL of FITC-**HepM-PLGA** was mixed with 900 μL of DMEM containing 10% FBS. The HepG2 cells and L02 cells were incubated with 200 μL of the incubation buffer in every well for 4 h. After the incubation buffer was discarded, the cells were trypsinized, collected by centrifugation at 1000 rpm for 2 min and washed thrice with PBS. Finally, the above HepG2 cells, BGC-823 cells, HeLa cells and MCF-7 cells were resuspended in 100 μL of PBS and the flow cytometry was used to obtain the data. All flow cytometry studies were conducted on an Image-StreamX multispectral imaging flow cytometer, and the data were analyzed using IDEAS software.

2.6 Validating the homologous targeting property of MCFM-PLGA with flow cytometry.

HepG2 cells, BGC-823 cells, HeLa cells and MCF-7 cells were seeded in a confocal cell culture dish and cultured for 24 h in 2 mL of DMEM with 10% FBS. After the supernatant was discarded, the HepG2 cells, BGC-823 cells, HeLa cells and MCF-7 cells were incubated with FITC-**MCFM-PLGA**. To prepare the incubation buffer, 100 μL of FITC-**MCFM-PLGA** was mixed with 900 μL of DMEM containing 10% FBS. The HepG2 cells and L02 cells were incubated with 200 μL of the incubation buffer in every well for 4 h. After the incubation buffer was discarded, the cells were trypsinized, collected by centrifugation at 1000 rpm for 2 min and

washed thrice with PBS. Finally, the above HepG2 cells, BGC-823 cells, HeLa cells and MCF-7 cells were resuspended in 100 μL PBS and the flow cytometry was used to obtain the data. All flow cytometry studies were conducted on an Image-StreamX multispectral imaging flow cytometer and the data were analyzed using IDEAS software.

2.7 Validating the homologous targeting property of RBCM-PLGA with confocal fluorescence imaging.

Human red blood cells (RBC) were used. The preparation procedure for RBC cell membrane was the same as the preparation process for HepG2 cell membranes. HepG2 cells, L02 cells, BGC-823 cells, HeLa cells and MCF-7 cells were seeded in a confocal cell culture dish and cultured for 24 h in 2 mL of DMEM with 10% FBS. After the supernatant was discarded, the HepG2 cells, L02 cells, BGC-823 cells, HeLa cells and MCF-7 cells were incubated with FITC-RBCM-PLGA. To prepare the incubation buffer, 100 μL of FITC-RBCM-PLGA was mixed with 900 μL of DMEM containing 10% FBS. HepG2 cells, L02 cells, BGC-823 cells, HeLa cells and MCF-7 cells were incubated with 200 μL of the incubation buffer in every well for 4 h. The incubation buffer was discarded, and the cells were washed three times with PBS (pH 7.4). Then, the cells were imaged immediately using a confocal microscope with an objective lens (× 63). Excitation of the probe-treated cells at 488 nm was performed using an argon laser, and the emitted light was collected with a META detector between 520 and 550 nm. The relative fluorescence intensity was measured by Zen software.

2.8 In vitro cytotoxicity assay.

The cytotoxicity of Dox-**HepM-PLGA** was examined by the MTT assay. HepG2 cells and L02 cells were seeded in 96-well microtiter plates and cultured for 24 h in 200 μL of Dulbecco's modified Eagle's medium (DMEM) with 10% FBS. After the supernatant was discarded, the HepG2 cells and L02 cells were incubated with Dox-**HepM-PLGA**, Dox-L02M-PLGA, Dox-PLGA and free Dox, respectively. To prepare the incubation buffer, 100 μL of Dox-**HepM-PLGA**, Dox-L02M-PLGA, Dox-PLGA or free Dox was mixed with 900 μL of DMEM containing 10% FBS. The HepG2 cells and L02 cells were incubated with 200 μL of incubation buffer in every well for 4 h. After the incubation buffer was discarded, 100 μL of MTT solutions (5 mg mL^{-1} in PBS) were added to each well away from light. After coincubation for 4 h, the remaining MTT solution was removed, and 100 μL of DMSO was added to each well to dissolve the formed formazan crystals. The absorbance was measured at 490 nm with an ELIASA

microplate reader. The experiment was repeated three times, and the data are shown as the mean± SD.

3. TEM image of PLGA nanoparticles and HepG2 cell membrane

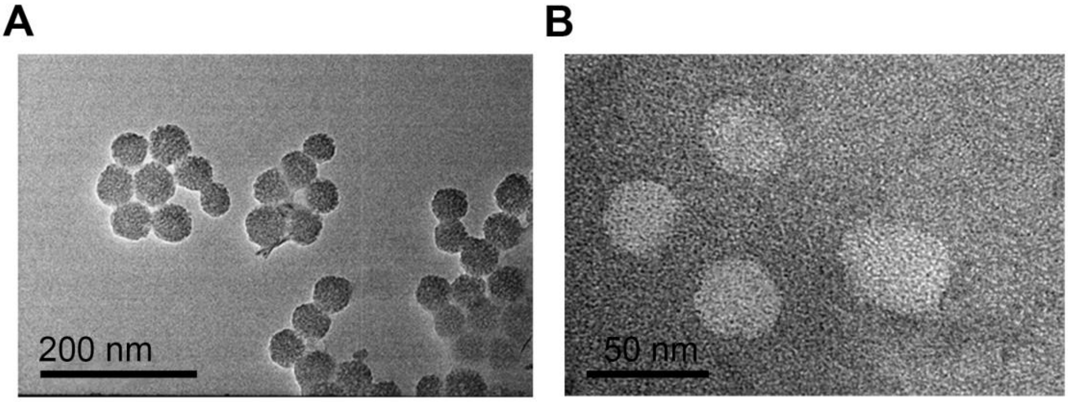

Figure S1. TEM image of (A)PLGA nanoparticles, (B) HepG2 cell membrane

4. Immunocompatibility assay of H22M-PLGA

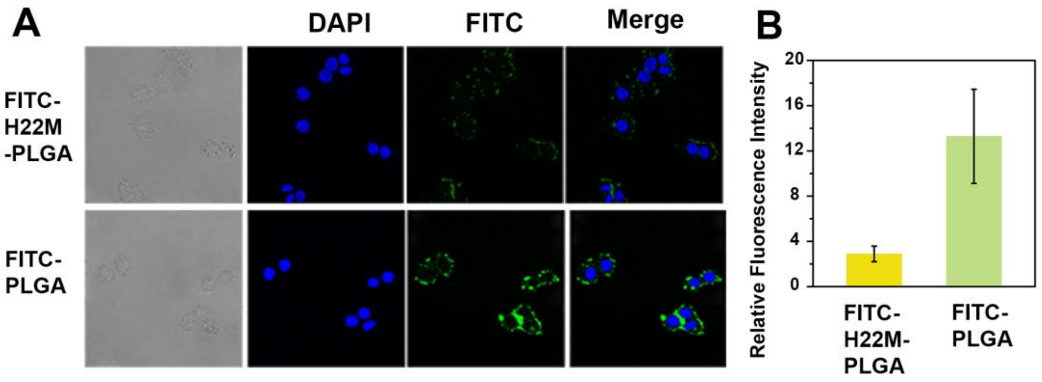

Figure S2. Immunocompatibility assay of H22M-PLGA. (A) Fluorescence images of particle internalization by RAW264.7 cells. The RAW64.7 cells were coincubated with FITC-H22M-PLGA nanoparticles and FITC-PLGA nanoparticles. (B) Quantitative fluorescence intensities corresponding to the images in (A). Scale bar: 50 μm.

5. Flow cytometry about the homologous targeting property of FITC-HepM-PLGA

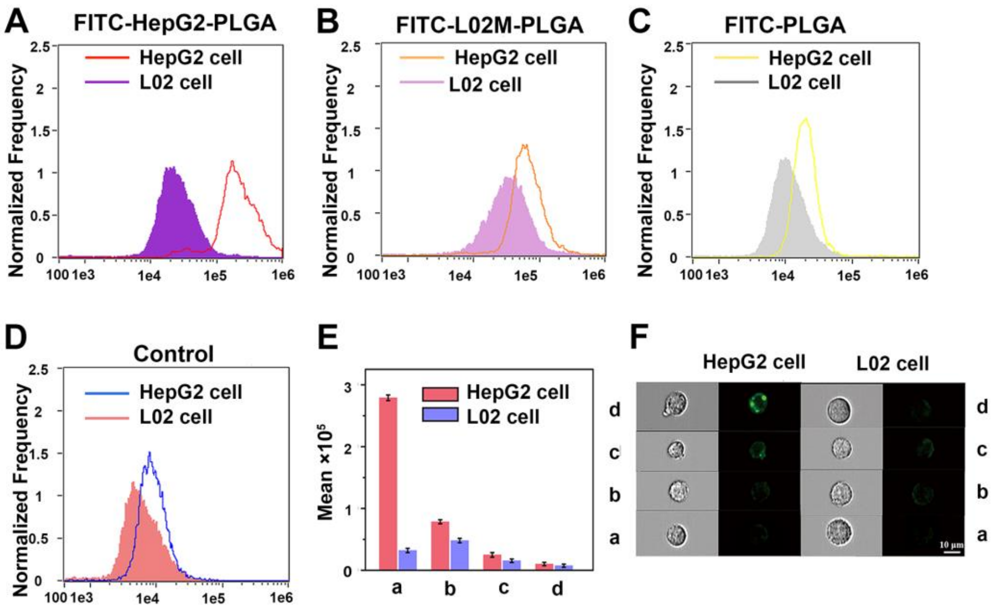

Figure S3. Flow cytometry analysis to validate the homologous targeting property of FITC-**HepM-PLGA** to HepG2 cells. (A) to (D) are the selective targeting of **HepM-PLGA** to HepG2 cells and other cells verified by flow cytometry. (E) Quantitative fluorescence intensities of results in (A) to (D). (F) Fluorescence images obtained with flow cytometry.

6. The targeting ability of HepM-PLGA toward HepG2 cells and other cancer cells

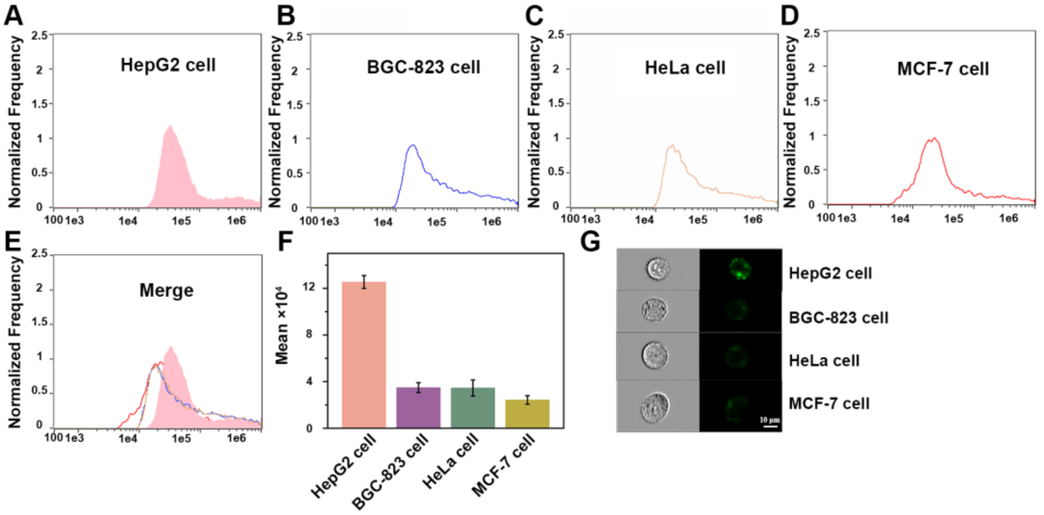

Figure S4. (A) to (E) Targeting ability of **HepM-PLGA** to HepG2 cells, BGC-823 cells, HeLa cells, and MCF-7 cell verified by flow cytometry. (F) Quantitative fluorescence intensities of results in (A) to (E). (G) Fluorescence images of HepG2 cells, BGC-823 cells, HeLa cells, and MCF-7 cell obtained with flow cytometry.

7. The targeting ability of MCFM-PLGA toward MCF-7 cells and other cancer cells

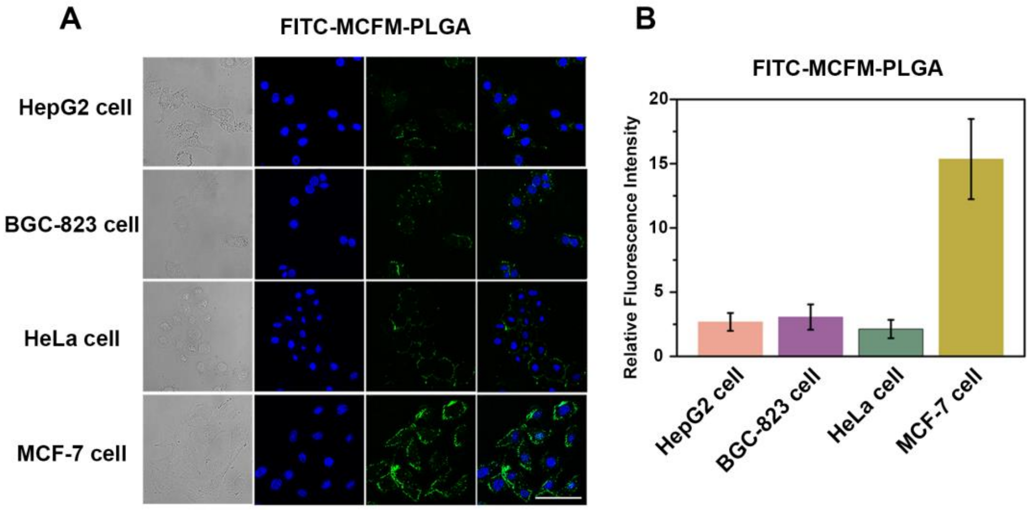

Figure S5. Fluorescence images of HepG2 cells (A), BGC-823 cells, HeLa cells and MCF-7 cells incubated with FITC-**MCFM-PLGA** nanoparticles. (B) Quantitative histograms of the fluorescence intensities of the images in (A). Scale bar: 75 μm.

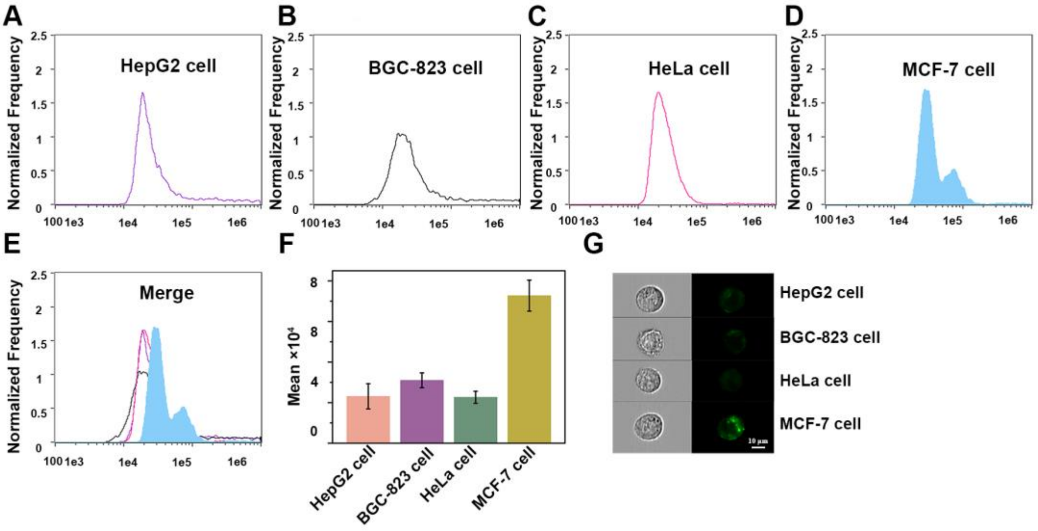

Figure S6. Flow cytometry analysis on the targeting ability of MCFM-PLGA (A) to (E) are the selective targeting of **MCFM-PLGA** to HepG2 cells, BGC-823 cells, HeLa cells, and MCF-7 cell verified by flow cytometry. (F) Quantitative fluorescence intensities of results in (A) to (E). (G) Fluorescence images obtained with flow cytometry.

8. The targeting ability of RBCM-PLGA

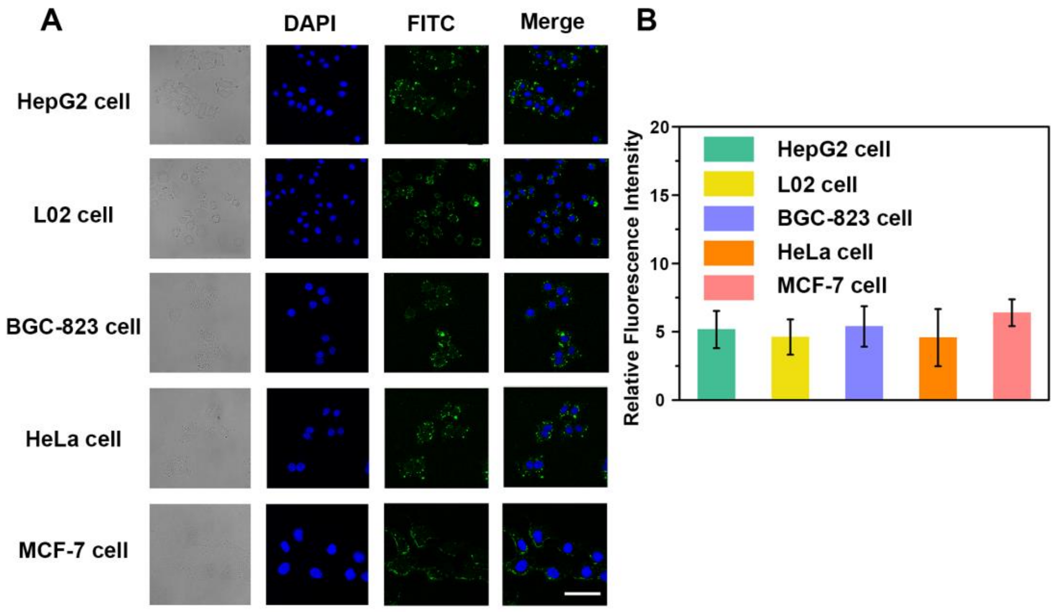

Figure S7. The targeting ability of **RBCM-PLGA** to HepG2 cells, L02 cell and other cancer cells. (A) Fluorescence images of HepG2 cells, L02 cell, BGC-823 cells, HeLa cells and MCF-7 cells incubated with FITC-**RBCM-PLGA** nanoparticles. (B) Quantitative histograms of the fluorescence intensities of the images in (A). Scale bar: 75 μm.

9. Dox loading content standard curve

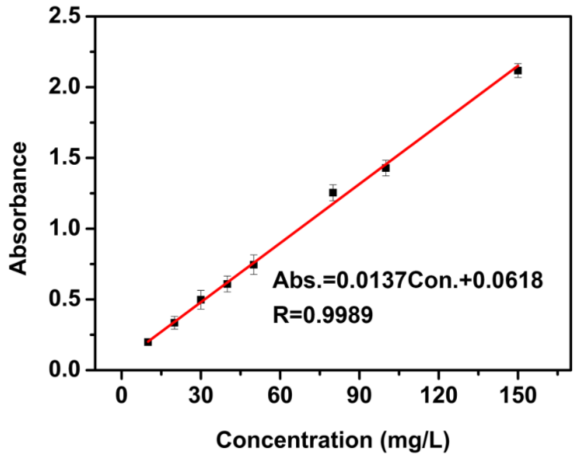

Figure S8 Dox loading content standard curve

10. MTT assay

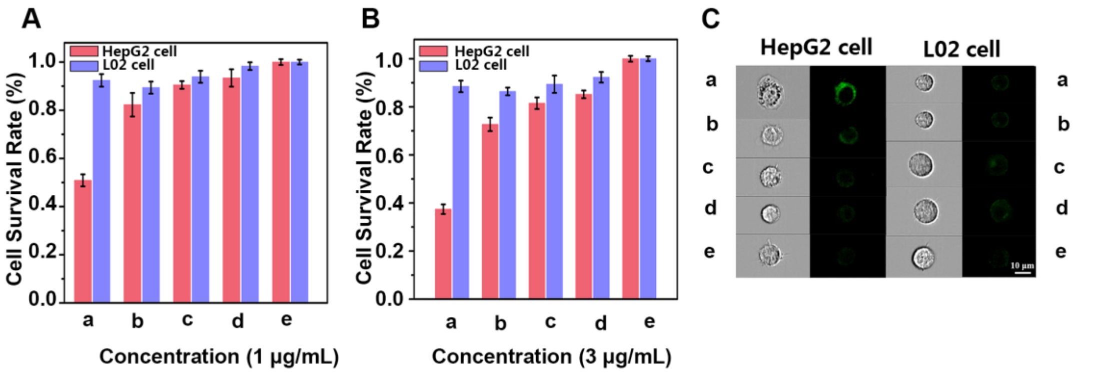

Figure S9 (A) and (B) MTT assay results. The concentration of Dox was 3 and 1 μg/mL. (C) Fluorescence images of HepG2 cells obtained with flow cytometry. a to e refer to HepG2 cells subjected to Dox-**HepM-PLGA**, Dox-L02M-PLGA, Dox-PLGA, free Dox and PBS, respectively.

11. In vitro therapeutic effect

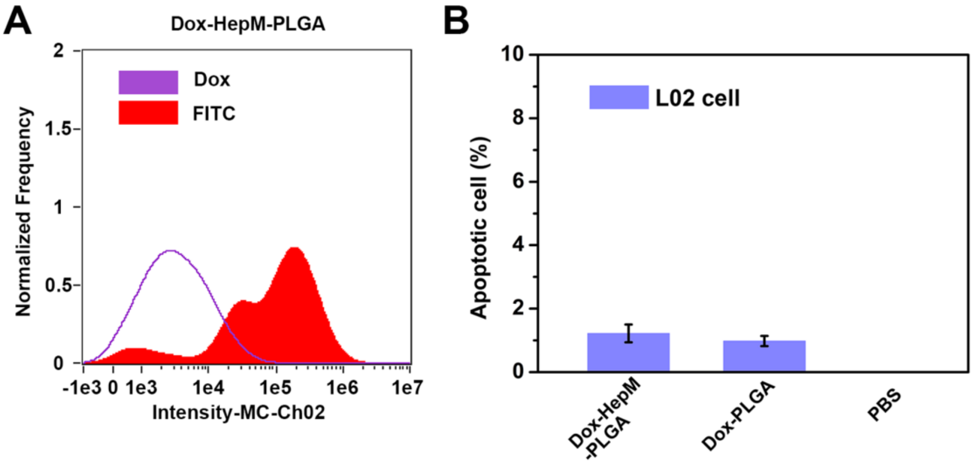

Figure S10 In vitro therapeutic effect. (A) The fluorescent intensity of Dox and FITC of HepG2 cells. The fluorescent intensity of Dox was collected after incubation with Dox-**HepM-PLGA** for 4 h. The fluorescent intensity of FITC was collected after incubation with Dox-**HepM-PLGA** for 4 h and then being stained with an Annex V-FITC/PI. (B) Apoptotic rate of L02 cells after incubation with Dox-**HepM-PLGA**, Dox-PLGA and PBS for 4 h and being stained with an Annex V-FITC/PI. Dox concentration: 38.88 μg/mg.

12. In vivo tumor image and antitumor effect after 3 days

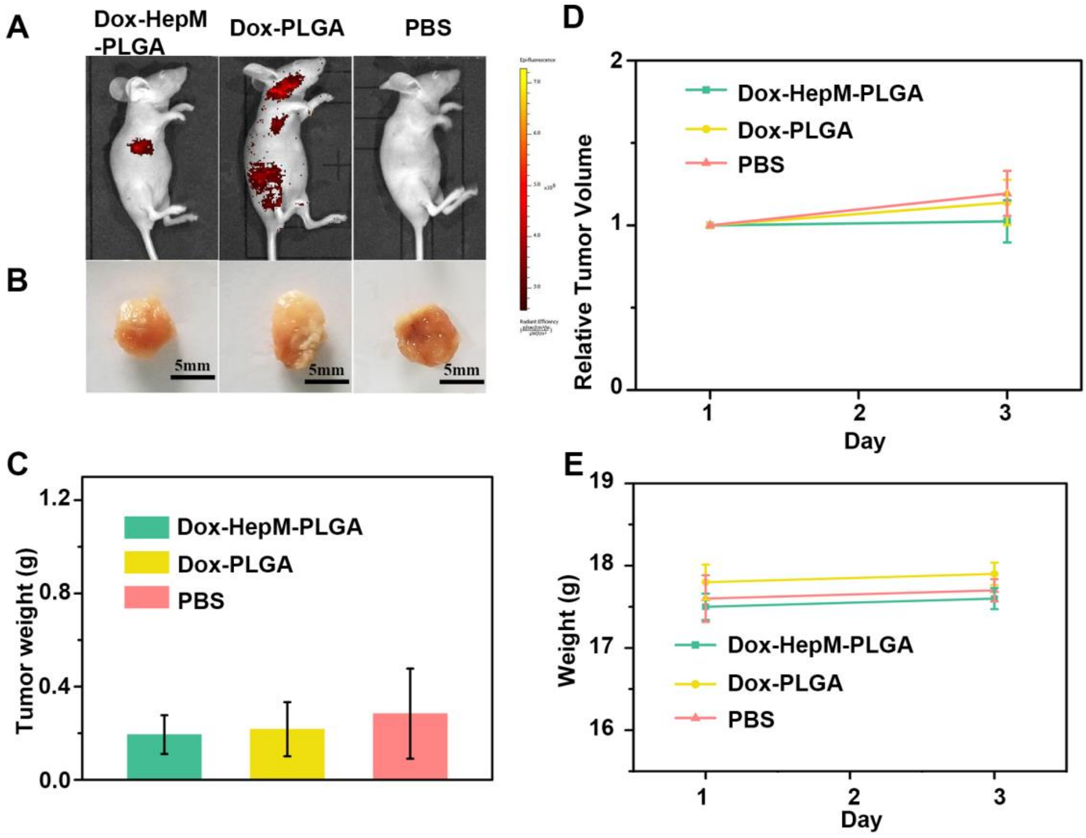

Figure S11 In vivo tumor image and antitumor effect after 3 days. (A) Fluorescence image of HepG2 tumor-bearing nude mice 3 days after the intravenous injection of Dox-**HepM-PLGA** and its counterparts. (B) Photos of the tumors extracted from the nude mice bearing the HepG2 tumor 3 days after the intravenous injection of Dox-**HepM-PLGA** and its counterparts. (C) Weights of the tumors extracted from the nude mice bearing the HepG2 tumor 3 days after the intravenous injection of Dox-**HepM-PLGA** and its counterparts. (D) Quantitative results of the HepG2 tumor relative volumes during chemotherapy. (E) Body weights of the nude mice during chemotherapy. All bars represent means ± s.d. *P ≤ 0.05, **P ≤ 0.01.

13. In vivo tumor image and antitumor effect after 7 days

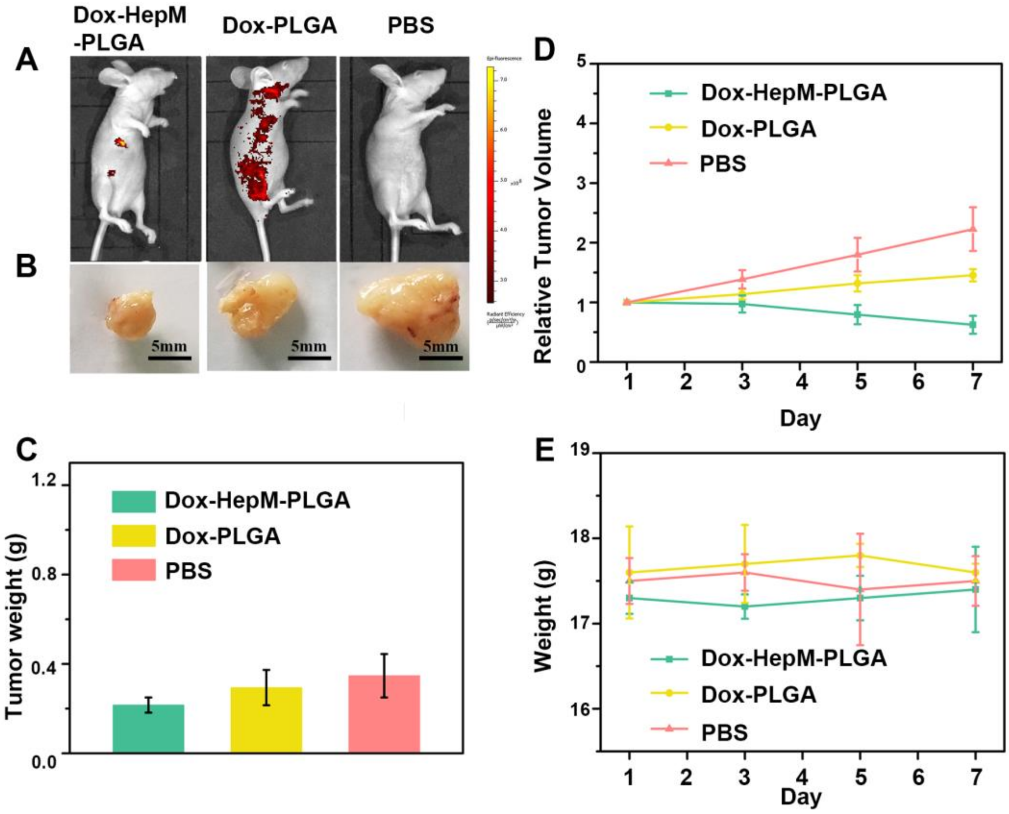

Figure S12 In vivo tumor image and antitumor effect after 7 days. (A) Fluorescence image of HepG2 tumor-bearing nude mice 7 days after the intravenous injection of Dox-**HepM-PLGA** and its counterparts. (B) Photos of the tumors extracted from the nude mice bearing the HepG2 tumor 7 days after the intravenous injection of Dox-**HepM-PLGA** and its counterparts. (C) Weights of the tumors extracted from the nude mice bearing the HepG2 tumor 7 days after the intravenous injection of Dox-**HepM-PLGA** and its counterparts. (D) Quantitative results of the HepG2 tumor relative volumes during chemotherapy. (E) Body weights of the nude mice during chemotherapy. All bars represent means ± s.d. $^{*}P \leqslant 0.05$, $^{**}P \leqslant 0.01$.

14. Fluorescence images of the major organs and tumors tissues.

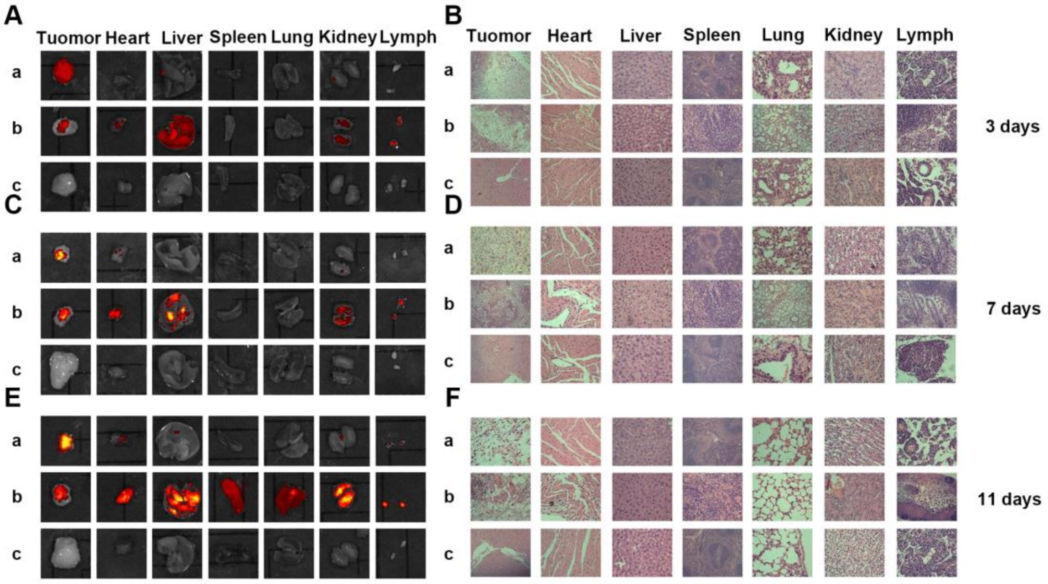

Figure S13 Fluorescence images of the major organs and tumors tissues. (A) Ex vitro fluorescence images of the major organs and (B) H&E staining of the tissue slices extracted from the HepG2 tumor-bearing nude mice 3 days after the intraperitoneal injection of Dox-**HepM-PLGA** and its counterparts. (C) Ex vitro fluorescence images of the major organs and (D) H&E staining of the tissue slices extracted from the HepG2 tumor-bearing nude mice 7 days after the intraperitoneal injection of Dox-**HepM-PLGA** and its counterparts. (E) Ex vitro fluorescence images of the major organs and (F) H&E staining of the tissue slices extracted from the HepG2 tumor-bearing nude mice 11 days after the intraperitoneal injection of Dox-**HepM-PLGA** and its counterparts. Scale bar: 50 µm. a to c refer to HepG2 tumor-bearing nude mice subjected to Dox-**HepM-PLGA**, Dox-PLGA and PBS, respectively.
